# Antiferroelectric-like Behavior in a Lead-Free Perovskite Layered Structure Ceramic

**DOI:** 10.1021/acs.inorgchem.2c02726

**Published:** 2022-12-06

**Authors:** Hangfeng Zhang, A. Dominic Fortes, Henry Giddens, Theo Graves Saunders, Matteo Palma, Isaac Abrahams, Haixue Yan, Yang Hao

**Affiliations:** † School of Electronic Engineering and Computer Science, Queen Mary University of London, Mile End Road, LondonE1 4NS, U.K.; ‡ STFC ISIS Facility, Rutherford Appleton Laboratory,Chilton Didcot, OxfordshireOX11 OQX, U.K.; § Department of Chemistry, Queen Mary University of London, Mile End Road, LondonE1 4NS, U.K.; ∥ School of Engineering and Materials Science, Queen Mary University of London, Mile End Road, LondonE1 4NS, U.K.

## Abstract

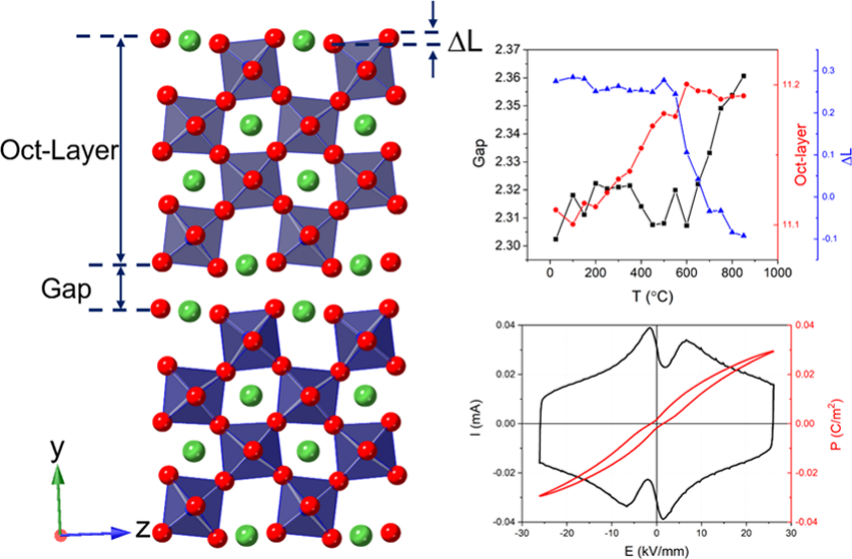

Antiferroelectric (AFE) materials have been intensively studied due to their potential uses in energy storage applications and energy conversion. These materials are characterized by double polarization–electric field (*P*–*E*) hysteresis loops and nonpolar crystal structures. Unusually, in the present work, Sr_1.68_La_0.32_Ta_1.68_Ti_0.32_O_7_ (STLT32), Sr_1.64_La_0.36_Ta_1.64_Ti_0.36_O_7_ (STLT36), and Sr_1.85_Ca_0.15_Ta_2_O_7_ (SCT15), lead-free perovskite layered structure (PLS) materials, are shown to exhibit AFE-like double *P*–*E* hysteresis loops despite maintaining a polar crystal structure. The double hysteresis loops are present over wide ranges of electric field and temperature. While neutron diffraction and piezoresponse force microscopy results indicate that the STLT32 system should be ferroelectric at room temperature, the observed AFE-like electrical behavior suggests that the electrical response is dominated by a weakly polar phase with a field-induced transition to a more strongly polar phase. Variable-temperature dielectric measurements suggest the presence of two-phase transitions in STLT32 at ca. 250 and 750 °C. The latter transition is confirmed by thermal analysis and is accompanied by structural changes in the layers, such as in the degree of octahedral tilting and changes in the perovskite block width and interlayer gap, associated with a change from non-centrosymmetric to centrosymmetric structures. The lower-temperature transition is more diffuse in nature but is evidenced by subtle changes in the lattice parameters. The dielectric properties of an STLT32 ceramic at microwave frequencies was measured using a coplanar waveguide transmission line and revealed stable permittivity from 1 kHz up to 20 GHz with low dielectric loss. This work represents the first observation of its kind in a PLS-type material.

## Abstract 2

Sr_1.68_La_0.32_Ta_1.68_Ti_0.32_O_7_ (STLT32), a lead-free perovskite layered structure (PLS) material, is shown to exhibit AFE-like double *P*−*E* hysteresis loops despite maintaining a polar crystal structure, which is the first observation of AFE-Like dielectric behavior in a PLS-type material. Variable temperature neutron diffraction reveals a phase transition accompanied by subtle structural changes in the layers, such as in the degree of octahedral tilting and changes in the perovskite block width and interlayer gap, associated with a change from non-centrosymmetric to centrosymmetric structures.

## 1 Introduction

Ferroelectric (FE) materials exhibit spontaneous polarization, which is switchable under an external electric field. DC electric field poling of FE materials results in a remanent polarization with a consequent piezoelectric response. Antiferroelectric (AFE) ordering occurs when adjacent dipoles are aligned antiparallel to each other leaving no net polarization. AFE phases may undergo field-induced transitions to polar FE phases, in which case piezoelectric properties can arise. Such field-induced transitions result in characteristic double hysteresis loops in polarization–electric field (*P*–*E*) measurements and open applications such as in high-field transducers, high-power energy storage capacitors, and energy conversion.^1−3^

The majority of commercial AFE materials, such as PbZrO_3_-based compounds, contain lead, and safety concerns on the use of lead as well as legislation now limit their continued commercial use. There has been much research interest in new lead-free antiferroelectric materials to replace the current lead-based systems. Most work in this area has focused on lead-free perovskites such as those based on AgNbO_3_,^4^ NaNbO_3_,^5^ and Bi_0.5_Na_0.5_TiO_3_-based compounds.^6,7^ Perovskite layered structure (PLS) ferroelectric materials, which show FE behavior at room temperature, have potential uses in high-temperature piezoelectric applications, such as in actuators and sensors for use in the automotive and gas industries.^8−10^ These materials are characterized by extremely high Curie points, *T*_C_, low dielectric loss, and a thermally stable piezoelectric coefficient, *d*_33_. Strontium tantalate (Sr_2_Ta_2_O_7_, ST) is a ferroelectric PLS material, with a remarkably low Curie point (*T*_C_) of −107 °C,^11^ much lower than those of many other ferroelectrics in the same family, such as Sr_2_Nb_2_O_7_ (*T*_C_ = 1342 °C),^11^ La_2_Ti_2_O_7_ (*T*_C_ = 1500 °C),^12^ and Nd_2_Ti_2_O_7_ (*T*_C_ = 1482 °C).^13^ It has been shown that the *T*_C_ of ST can be increased by A-site substitution with Ca^2+^, B-site substitution by Nb^5+^,^14^ or A/B-site cosubstitution by La^3+^ and Ti^4+^ in Sr_2–*x*_La_*x*_Ta_2–*x*_Ti_*x*_O_3_.^15^ In Sr_2–*x*_La_*x*_Ta_2–*x*_Ti_*x*_O_3_, it was found that at relatively low levels of substitution (*x* ≤ 0.1), *T*_C_ was increased to 200 °C, leaving the material FE at room temperature, while the structure of ST at room temperature is nonpolar in space group **Cmcm**,^16^ cooling to below −107 °C, leads to a reduction of symmetry and the stabilization of a polar phase in space group *Cmc*2_1_, giving rise to spontaneous polarization along the *c*-axis.^17^ It should be noted that there is some argument in the literature regarding the structure of the paraelectric (PE) phase of ST at room temperature. Based on superlattice reflections in electron diffraction data, Yamamoto et al. claimed that the structure below 170 °C is actually monoclinic in space group *P*2_1_/*m*,^18^ but no structural details of this model were presented. Relaxor behavior has been reported to occur at −223 °C in Sr_2_Ta_2_O_7_. However details of the structural changes associated with this behavior remain unclear.^19^ One of the aims of the present work is to establish the nature of this transition through a detailed structural study.

In this work, we investigate the electrical and structural characteristics of the double- and single-substituted ST compounds, Sr_1.68_La_0.32_Ta_1.68_Ti_0.32_O_7_ (STLT32), Sr_1.64_La_0.36_Ta_1.64_Ti_0.36_O_7_ (STLT36), and Sr_1.85_Ca_0.15_Ta_2_O_7_ (SCT15). The structural and electrical properties of these materials are examined as functions of temperature using high-resolution powder neutron diffraction, piezoresponse force microscopy, and impedance analysis. Unusually, despite possessing a polar structure at room temperature, STLT32 and STLT36 exhibit AFE-like behavior with double *P–E* hysteresis loops, the first observation of its kind in lead-free PLS materials. The dielectric properties remain stable up to mm-wavelengths and open up possible applications in 5G communication devices as dielectric resonators.

## 2 Experimental Section

STLT32, STLT36, and SCT15 ceramics were synthesized by a conventional solid-state method. Stoichiometric amounts of SrCO_3_ (Aldrich, 99.9%), CaCO_3_ (Sigma-Aldrich, 99.5%), La_2_O_3_ (Alfa Aesar, 99.9%), Ta_2_O_3_ (Alfa Aesar, 99.85%), and TiO_2_ (Aldrich, 99.8%) were ball-milled in nylon pots with zirconia balls and ethanol as a dispersant. The ball milling process was carried out for 4 h at a speed of 170 rpm, and the resulting slurry dried at 80 °C. In each case, the dry powder was sieved using a 250 μm sieve, transferred to an alumina crucible, and then calcined in a furnace at 1000 °C for 2 h. After cooling, the powder was remilled in ethanol, dried, and sieved again. The resulting powder was pressed uniaxially into 20 mm diameter pellets at a pressure of ca. 150 MPa and sintered at 1600 °C for 2 h.

X-ray powder diffraction (XRD) data were collected on crushed ceramic powder using a PANalytical X’Pert Pro diffractometer fitted with an X’Celerator detector. Data were collected with Ni-filtered Cu Kα radiation (λ = 1.5418 Å), at room temperature, in a flat plate θ/θ geometry over the 2θ range 5–120° with a step width of 0.0334° and an effective count time of 50 s per step. For the STLT32 composition, powder neutron diffraction data were collected on the high-resolution powder diffractometer (HRPD) at the ISIS Facility, Rutherford Appleton Laboratory, UK. The sample was placed in an aluminum alloy slab geometry sample container with internal dimensions 18 mm × 23 mm perpendicular to the incident beam and 10 mm depth parallel to the beam. The sample was held between vanadium foil windows, and all exposed Al and steel components of the cell were masked with Gd and Cd foils. A RhFe thermometer and a cartridge heater, which was embedded in the sample holder frame, were used to control temperature. The exchange gas was completely evacuated from around the sample, and data were collected at selected temperatures from 25 to 850 °C. XRD and neutron data were modeled by Rietveld analysis using the GSAS suite of programs^20^ with orthorhombic models in space groups *Cmc*2_1_^21^ or **Cmcm**.^16^ Scanning electron microscopy (SEM, FEI Inspect-F Oxford) was used to examine the morphology of the ceramic fracture surface, and elemental analysis was carried out on the carbon-coated surface using energy-dispersive X-ray analysis (EDX). High-temperature simultaneous thermogravimetric analysis and differential scanning calorimetry (TGA–DSC) were carried out from room temperature to 1000 °C, at a ramp rate of 5 °C min^–1^, using an STA 499 F3 (NETZSCH, Germany). Piezoresponse force microscopy (PFM) was performed on an AFM system (Bruker Dimension Icon, US) using an SCM-PIT-V2 conductive probe (Bruker, US).

The surfaces of ceramic pellets were coated with silver paste (Gwent Electronic Materials Ltd. Pontypool, U.K.) and heated at 300 °C for electrical measurements. Pellets were ground to a rectangular shape of approximate dimensions 4 mm × 4 mm × 0.3 mm. For high-temperature dielectric measurements, samples were coated with a Pt paste (Gwent Electronic Materials Ltd., Pontypool, U.K.) and fired at 1000 °C for 10 min. The temperature dependencies of dielectric permittivity and loss were measured using an LCR meter (Agilent 4284a) from room temperature to 1000 °C at selected frequencies. Current density–electric field (*I*–*E*) and polarization–electric field (*P–E*) loops were measured at room temperature using a ferroelectric hysteresis measurement tester (NPL, UK). The electric voltage was applied in a triangular waveform.^22^

The microwave dielectric properties for STLT32 were measured from 2 to 18 GHz by depositing a coplanar waveguide (CPW) transmission line onto the surface of the ceramic samples through a thermally evaporated silver coating. The dielectric properties were retrieved from the propagation constant via measurements of the two-port S-parameters using a PNA-L N5230C vector network analyzer with a fully calibrated GSG microprobe 525. The conductive losses were removed through a reference measurement of the same device on an Al_2_O_3_ substrate. The lengths of the CPW transmission lines were 1 and 5 mm, and the thickness of the silver coating was 1 μm.

## 3 Results and Discussion

Figure S1 shows a comparison of fits in space groups **Cmcm** and *Cmc*2_1_ to the X-ray powder diffraction patterns of STLT32, STLT36, and SCT15. The SCT composition was found to be phase pure, but in both the STLT compositions, a minor secondary five-layer perovskite phase was fitted using the model for SrLa_4_Ti_5_O_17_ in space group *Pnnm*.^23^ Stacking faults have previously been identified in Sr_2_(Ta_1–*x*_Nb_*x*_)_2_O_7_ ceramics, resulting in the coexistence of different layer stacking sequences in these PLS materials. They form through the elimination of oxygen vacancies and as a consequence lower the dielectric loss.^14^ For each composition, the X-ray patterns are well fitted by both polar and nonpolar models with no clear features observed to distinguish the models and similar reliability factors.

**Figure 1:**
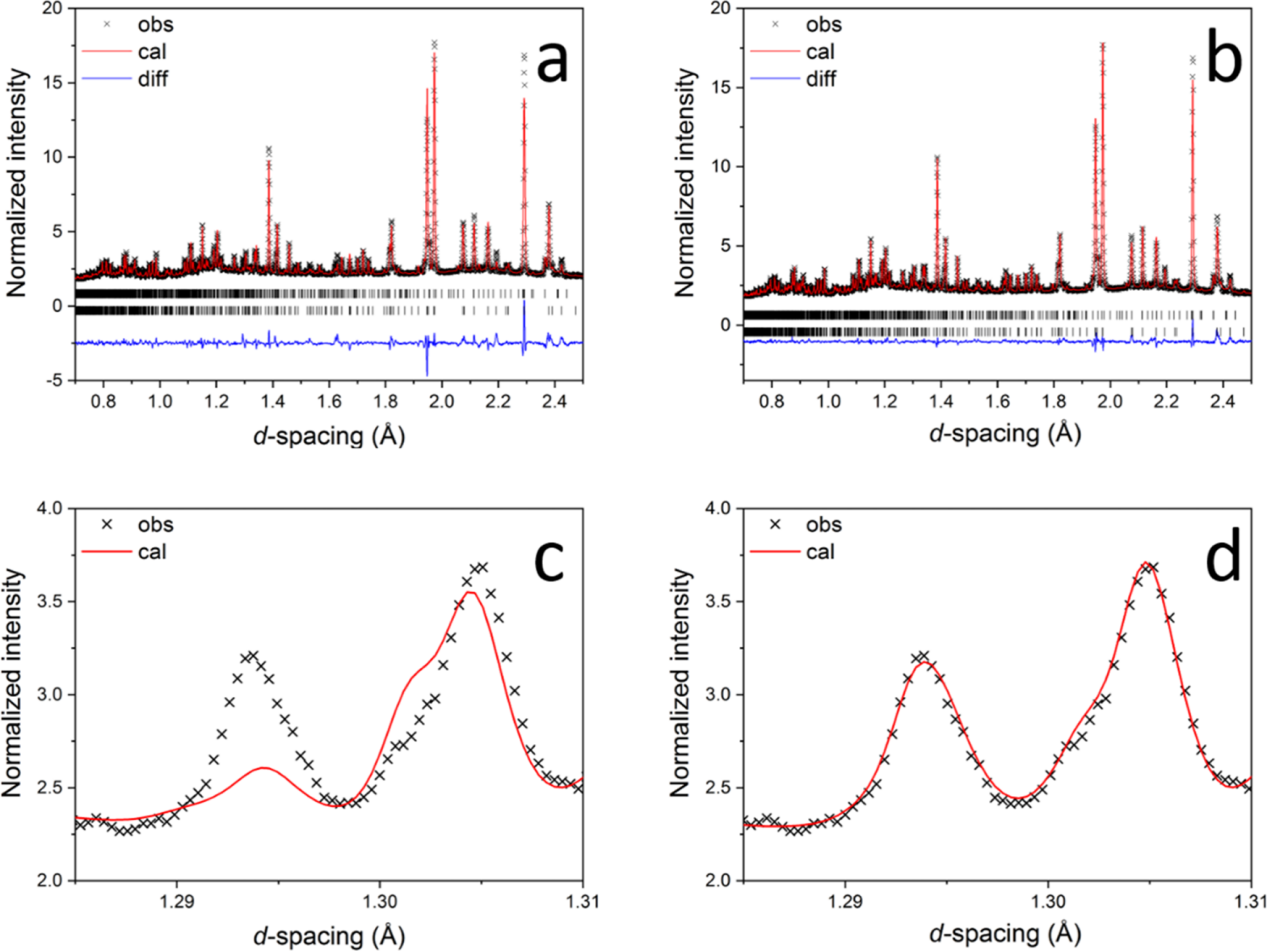
Fitted neutron diffraction profiles for STLT32 at 25 °C using (a) **Cmcm** and (b) C*mc*2_1_ models with a five-layer secondary phase in space group *Pnnm* included in both cases; details of the fits around 1.3 Å are given in panels (c) and (d), respectively. Reflection positions are indicated by markers.

Compared to XRD, high-resolution neutron diffraction offers higher accuracy in the determination of oxygen positions in the presence of heavier cations and allows for subtle structural features to be characterized. Figure 1a,b show the high-resolution neutron diffraction patterns of STLT32 fitted using the nonpolar **Cmcm** and polar *Cmc*2_1_ models, respectively, with refinement parameters summarized in Tables S1 and S2. Although all of the diffraction peaks are fitted in both models, a significantly better *R*-factor (2.35%) was obtained using the *Cmc*2_1_ model, compared to a value of 4.18% with the **Cmcm** model. The (154) peak at 1.293 Å is clearly better fitted using the polar phase model (Figure 1c,d). As discussed below, this particular peak changes intensity with temperature and can be used to distinguish the nonpolar and polar phases. The basic reason for this is a difference in structure factors for this reflection in the two models. There are other reflections, which also show significant differences in structure factors, but due to overlap with other reflections, these are less obvious than those for the (154) peak. Surface and cross-section SEM images of STLT32 samples (Figure S2) show a dense ceramic with platelike grains ∼3 μm in length and 0.5 μm in thickness. Figure S3 shows the results of an EDX analysis on an STLT32 sample and suggests that the composition is slightly rich in Ta compared to the theoretical composition.

**Figure 2:**
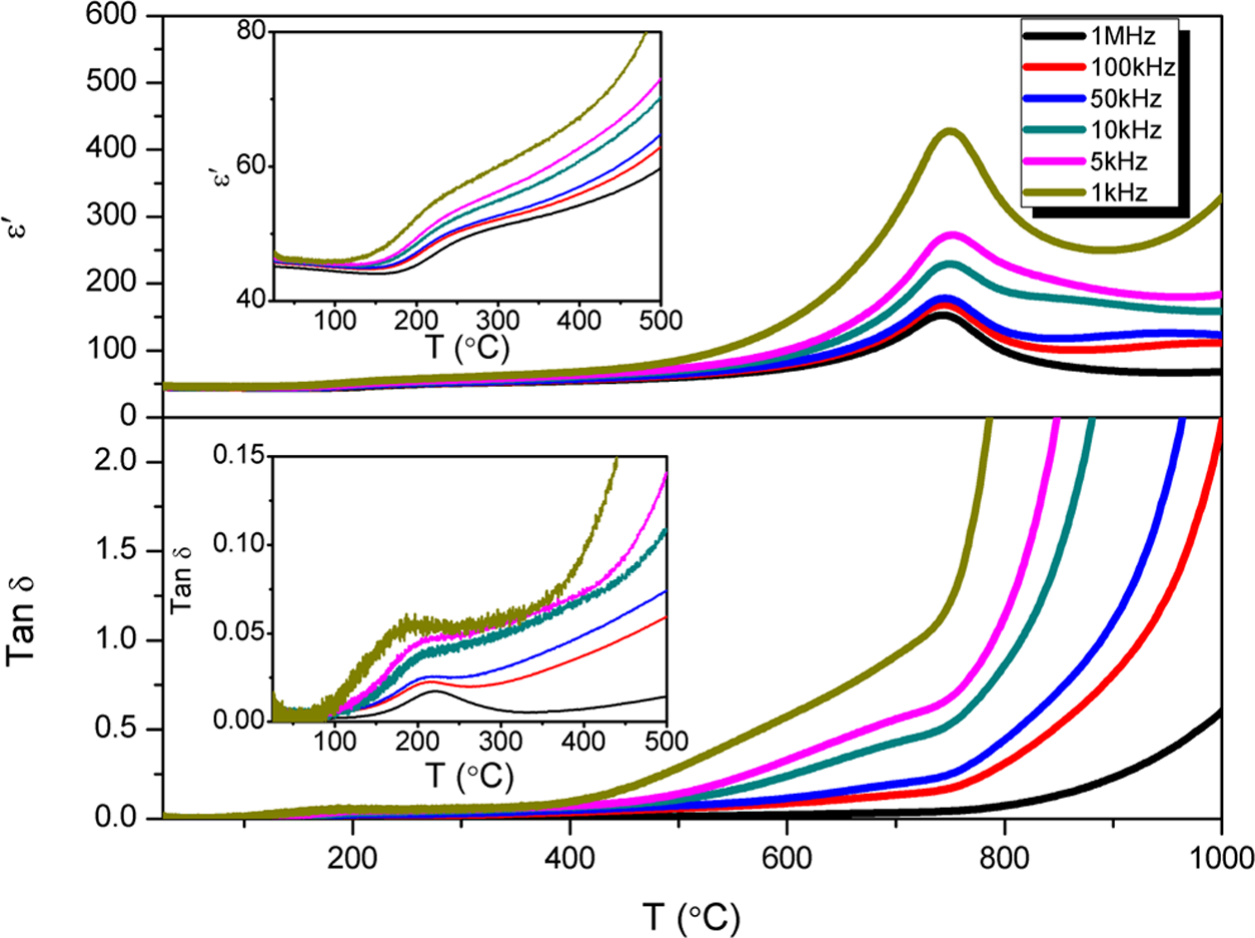
Thermal dependencies of dielectric permittivity and loss tangent for STLT32.

Figures 2 and S4 show the temperature dependencies of dielectric permittivity and loss tangent for STLT32, STLT36, and SCT15 at selected frequencies. The five-layer secondary phase in the STLT compositions is thought to contribute little to the overall permittivity and loss. Similar five-layer-based materials such as SrLa_4_Ti_5_O_17_ are nonferroelectric and used for microwave applications due to their low dielectric permittivity and low loss up to microwave frequencies.^23^ A clear dielectric permittivity peak is seen in the spectra for STLT32 and STLT36 at ca. 750 and 790 °C, respectively, with a corresponding curve upward in the loss tangent, which is observed to be frequency-independent, suggesting an FE to PE phase transition, i.e., the Curie point. Close inspection of the plots in the low-temperature range (Figures 2 and S4a) reveal an anomaly in dielectric permittivity at ca. 220 and ca. 250 °C for STLT32 and STLT36, respectively, with the corresponding loss peaks shifting to higher temperatures at higher frequencies. The frequency dependence of this dielectric feature indicates a possible diffusive or relaxor-type phase transition. SCT15 shows two anomalies in dielectric permittivity between 100 and 200 °C, each of which is suggested to correspond to a phase transition. Three dielectric anomalies have been reported in the dielectric permittivity spectra of a single crystal Sr_2_Ta_2_O_7_, at ca. −223, −118, and 167 °C, denoted as *T*_3_, *T*_2_ or *T*_C_, and *T*_1_, respectively,^19^ where the anomaly at the lowest temperature, *T*_3_, shows a similar dielectric dispersion to that seen in the present work for the STLT compositions.

**Figure 3:**
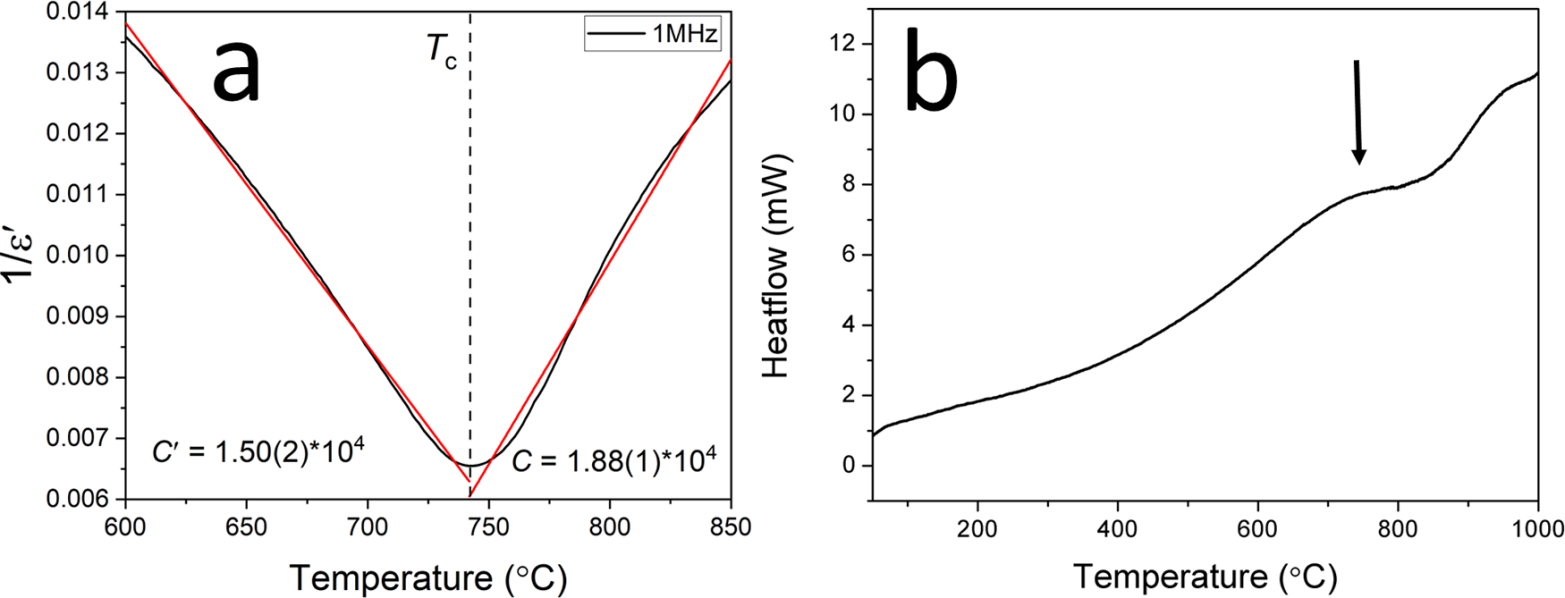
(a) Temperature dependence of reciprocal of dielectric permittivity for STLT32 and Curie–Weiss fitting of the reciprocal of permittivity before and after *T*_C_. (b) DSC thermogram for STLT32.

The phase transition at 750 °C was investigated by the Curie–Weiss law using the following equation
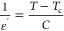
1where *C* and *T*_C_ are the Curie constant and Curie point, respectively. Figure 3a shows the reciprocal of dielectric permittivity for STLT32 fitted using the Curie-Weiss law, where *C’* and *C* are the Curie constants below and above the Curie point, respectively. The *C’*/*C* ratio is less than 4, which indicates that the transition at 750 °C is second-order.^24^ A DSC thermogram for STLT32 is shown in Figure 3b and shows a weak feature with an onset temperature of ca. 750 °C. This feature coincides with the dielectric permittivity peak seen in Figure 2 and is consistent with a second-order transition between FE and PE phases.

**Figure 4:**
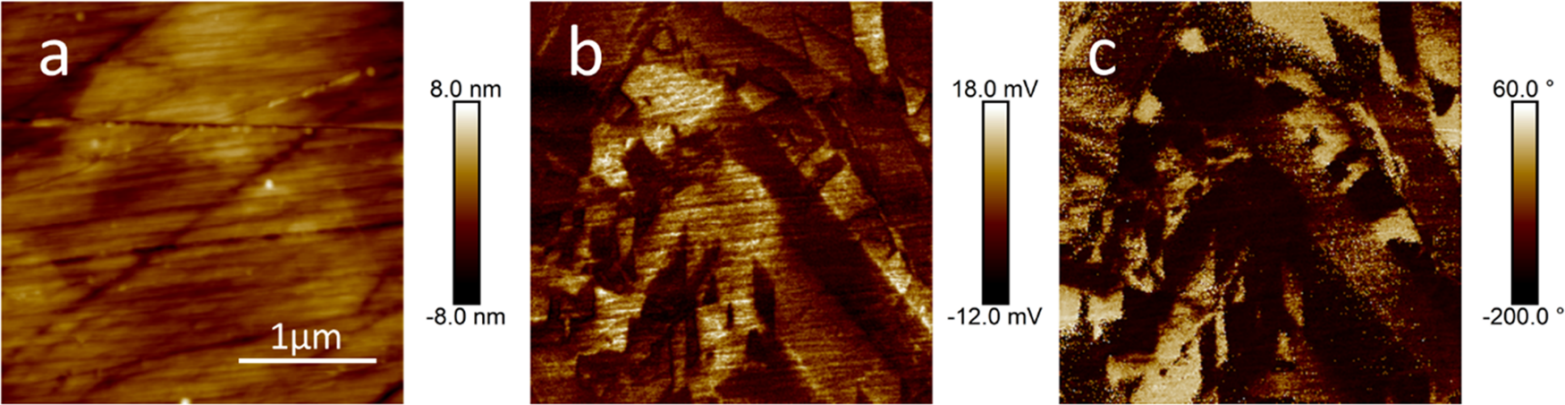
(a) Topographic, (b) magnitude, and (c) phase PFM images of an STLT32 ceramic sample.

Figure 4 shows 3 μm × 3 μm PFM images of an STLT32 ceramic sample. Surface roughness obtained from the topographic image was below 20 nm after polishing (Figure 4a). The appearance of the magnitude and phase images (Figure 4b,c) were similar but differ to the topographic image as their signal corresponds to the domain structures rather than surface roughness. The color contrast in the magnitude and phase images represents the different piezoresponses of the FE domains with different polar directions in the material. Micron-sized domains are evident as dark/light regions randomly distributed in the scanned area. The PFM results lend further support to an average ferroelectric phase.

**Figure 5:**
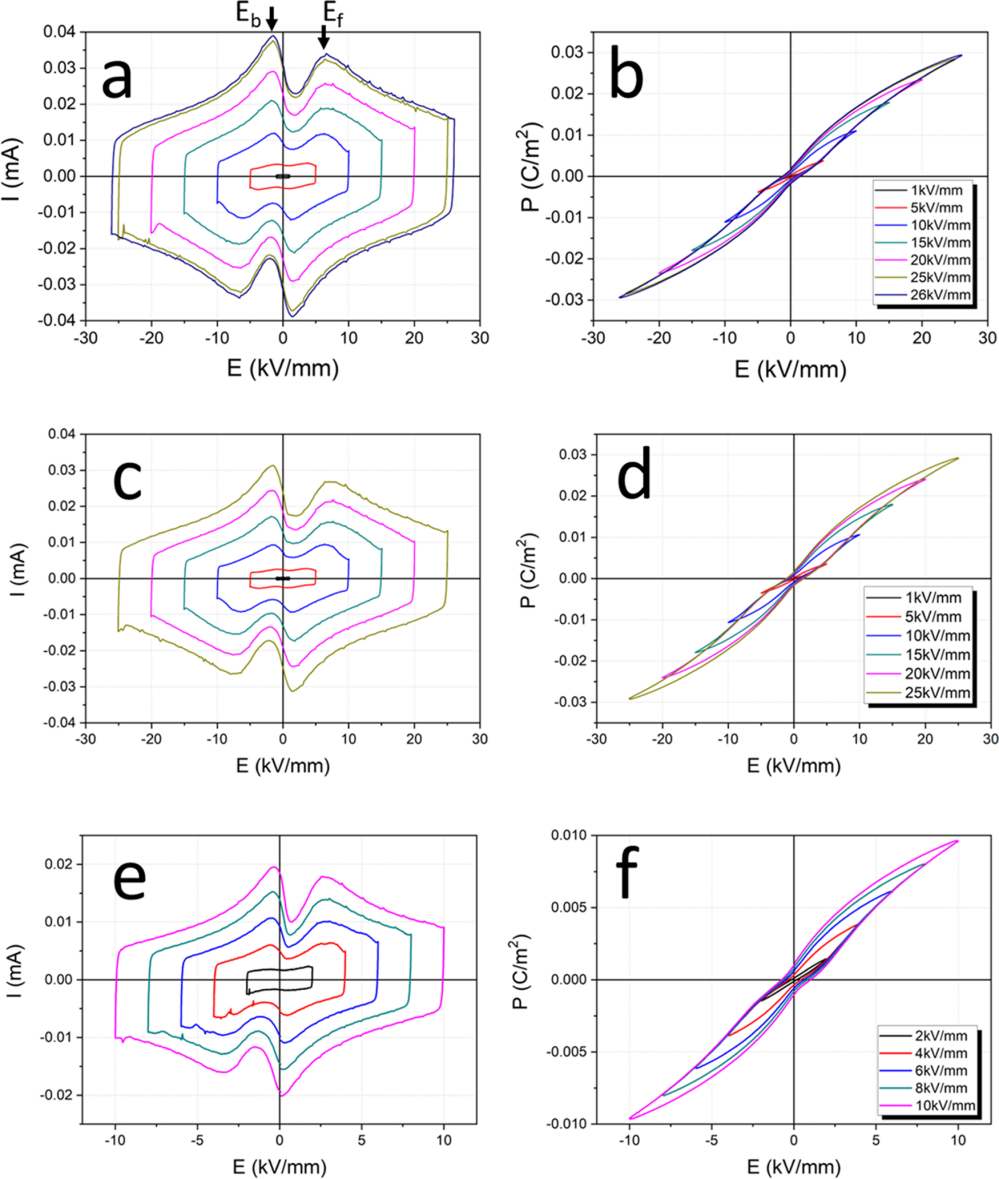
(a, c, e) *I*–*E* and (b, d, f) *P*–*E* loops for (a, b) STLT32, (c, d) STLT36, and (e, f) SCT15 measured at 10 Hz.

Figure 5 shows *I–E* and *P–E* loops for the studied compositions at 10 Hz. Four current peaks are seen in the *I–E* data, viz., two sharper peaks for STLT32 at ca. ±1.5 kV mm^–1^ corresponding to the backward electric field, *E*_b_, and two broader peaks corresponding to the forward electric field, *E*_f_, at ca. ±6.5 kV mm^–1^. The four current peaks in the *I*–*E* loop and narrow double hysteresis *P–E* loops suggest AFE-like behavior,^25^ the first time such behavior has been observed in a lead-free PLS system. The results indicate a reversible field-induced phase transition in STLT32, STLT36, and SCT15 at room temperature. XRD patterns for STLT32 before and after poling (Figure S5) are indistinguishable and support the proposition that the field-induced transition is fully reversible. The occurrence of this behavior in SCT15, which is a single-phase four-layer composition, supports the proposition that this AFE-like behavior is intrinsic to the four-layer structure. In conventional antiferroelectrics, such as AgNbO_3_, NaNbO_3_, and PbZrO_3_, double hysteresis loops are only observed at high electric fields, and often in bulk ceramics electrical breakdown occurs before these high fields can be reached.^5,26,27^ The double hysteresis loops in STLT32 occur consistently over both low and high fields up to the observed breakdown field of 26 kV mm^–1^. The narrowness of the hysteresis loops with low remnant polarization indicates low energy loss during charge–discharge cycling. *I–E* and *P–E* data measured at elevated temperatures confirm that this AFE-like behavior persists up to at least 200 °C (Figure S6). Figure S7 shows that four current peaks are seen in *I*–*E* loops with consistent double hysteresis loops for STLT32 at different frequencies and supports the proposition that the double hysteresis *P*–*E* loops observed in Figure 5 are indeed an intrinsic property of the material.

Although the neutron diffraction and PFM results suggest that the STLT32 system is ferroelectric at room temperature, the AFE-like electrical behavior may be associated with a weakly polar intermediate phase. Such behavior is not unknown in AFE systems. For example, in AFE compositions in the PbZr_1*–x*_Ti_*x*_O_3_ (PZT) system, an incommensurate structure is observed in transmission electron microscopy (TEM) images and it has been suggested that the incommensurate phase results from competition between long-range FE and AFE orders.^26^ Similarly, double hysteresis loops seen in the *P–E* data at temperatures far below *T*_C_, for Ba_4_Sm_2_Ti_4_Nb_6_O_30_ and Ba_4_Eu_2_Ti_4_Nb_6_O_30_,^28^ have been suggested to be caused by field-induced transitions between polar commensurate and nonpolar incommensurate phases. While an incommensurate phase has not been identified in STLT32, TEM results for strontium tantalate Sr_2_Ta_2_O_7_ and the Sr_2_Ta_2–*x*_Nb_*x*_O_7_ solid solution do show evidence of such an incommensurate phase.^14,18^ Thus, it is suggested here that in STLT32, a field-induced transition occurs between a weakly polar phase and a more strongly polar phase.

Figure S8 shows the normalized dielectric permittivity change in STLT32 with DC bias field. With increasing applied field, the dielectric permittivity increases up to a field of ca. 1.5 kV mm^–1^ but then decreases at higher fields. This contrasts with the situation in normal ferroelectric or dielectric materials, where dielectric permittivity generally decreases with increasing applied DC field due to the alignment of the dipoles with the field direction and consequent restriction of dipole activity. The unusual field behavior of STLT32 suggests a phase transition from a weakly polar phase to a more strongly polar phase, which would indicate that the weakly polar phase must be present in the virgin material. Typically, FE PLS materials do not easily switch under an applied electric field due to their extremely high coercive fields.^29−31^ Therefore, it is the weakly polar phase that readily shows the observed reversible field-induced transitions that dominate the electrical response.

**Figure 6:**
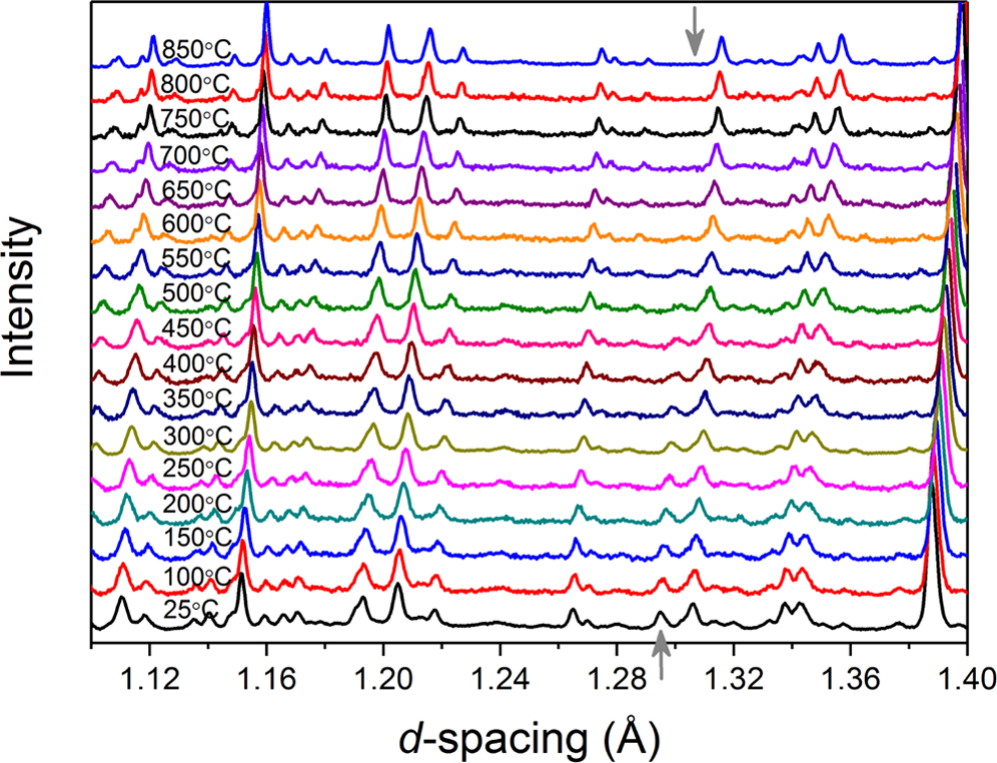
Detail of neutron diffraction profiles for STLT32 as a function of temperature.

**Figure 7:**
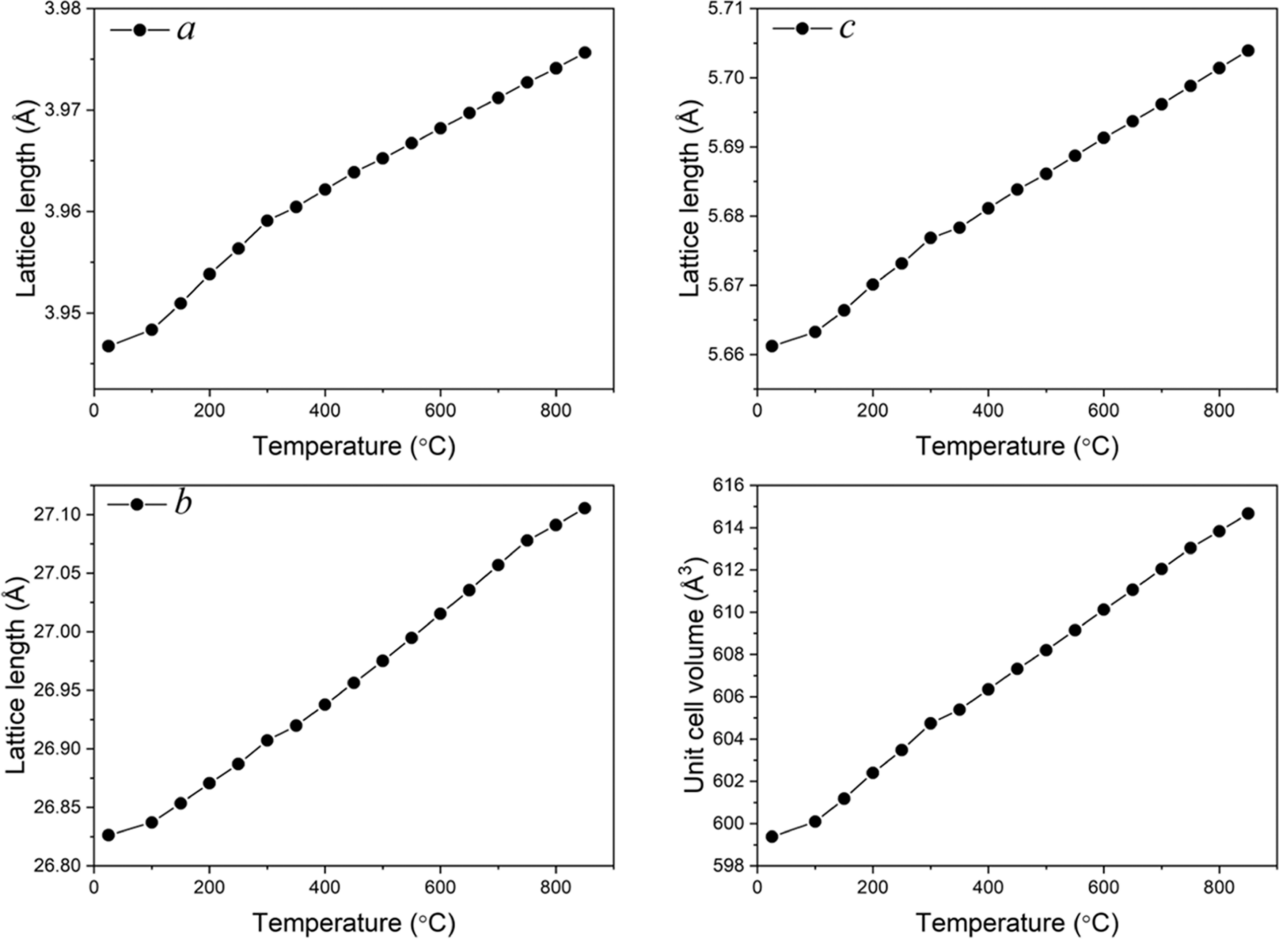
Temperature variation of lattice parameters *(a, b*, *c*) and unit cell volume in STLT32.

Figure 6 shows the thermal variation of the neutron diffraction pattern for STLT32. The data show an expected shift of the diffraction peaks to higher *d*-spacing due to thermal expansion of the lattice. With increasing temperature, the (154) peak at ca. 1.293 Å gradually decreases in intensity and vanishes at 750 °C, and as discussed above, its intensity can be used to distinguish between the polar *Cmc*2_1_ (high intensity) and the nonpolar **Cmcm** phases (low intensity). The temperature of this transition is consistent with the dielectric spectra (Figure 2) and the DSC results (Figure 3). Figure 7 shows the thermal variation of the refined lattice parameters and unit cell volume, which are summarized in Table S3. A clear change is seen in the slope of all parameters at around 300 °C, which correlates with the anomaly found in the dielectric spectra at around the same temperature. In addition, the *b*-axis data show a change in slope at 750 °C, which again agrees with the transition seen in the electrical and also the thermal analysis data and is attributed to the polar to nonpolar phase transition. The thermal variation of the *c*/*a* lattice parameter ratio is shown in Figure S9. It shows a steady decrease on heating up to 350 °C, followed by a steady increase with further increase in temperature. The polarization in space group *Cmc*2_1_ primarily arises along the *c*-axis. Given that the thermal expansions along the *a*- and *c*-crystallographic axes are similar (Figure 7), the initial decrease in the *c*/*a* ratio would be consistent with decreasing polarization with increasing temperature and inversely increasing polarization with increasing temperature above 350 °C. The abrupt change at 350 °C may be associated with a transition from weakly polar to more strongly polar states but is slightly above the transition temperatures seen in the dielectric spectra. The difference is likely due to the diffuseness of the feature in the dielectric permittivity spectra and the different heating rates used in these experiments.

**Figure 8:**
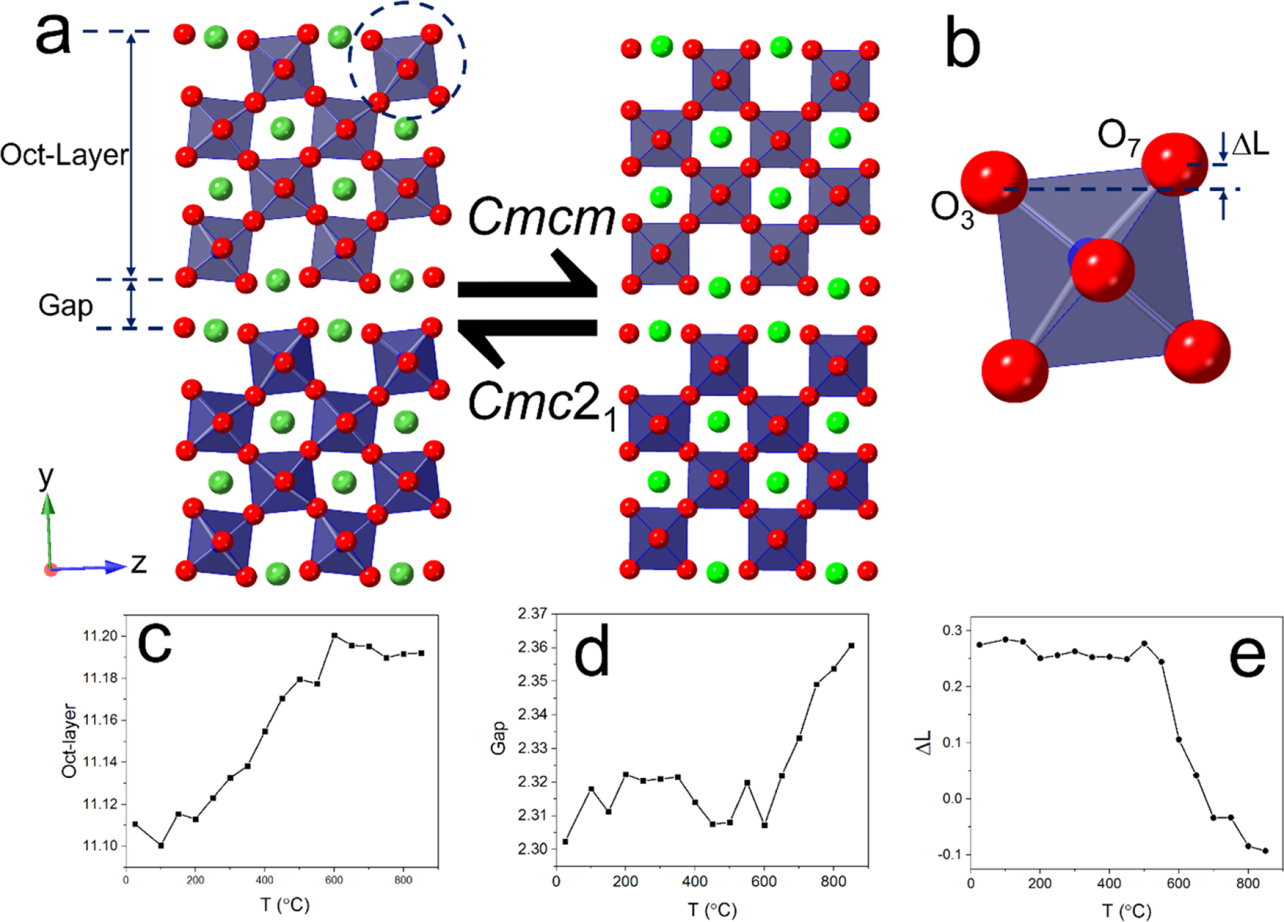
(a) Crystal structure of STLT32 viewed in the *bc* plane with the circled octahedron enlarged and shown in panel (b). Temperature variation of (c) octahedral layer width, (d) interlayer gap, and (e) Δ*L* distance derived from neutron diffraction analysis.

The thermally induced transition from polar to nonpolar phases may be followed by considering details of the layer structure of STLT32. Figure S10 shows the variation in M–O–M (M = Ta/Ti) bond angles. Ta1–O2–Ta2 increases with increasing temperature, while Ta1–O6–Ta2 increases up to around 500 °C where it reaches a maximum 180° angle. Ta1–O1–Ta1 remains fairly constant apart from a fluctuation around 600 °C. The increasing M–O–M bond angles indicate a gradual reduction in octahedral tilting as the system evolves from polar to nonpolar structures. Clearer evidence for this transition can be obtained by examining some general structural features. The ideal ABO_3_ perovskite structure may be considered as an infinite set of octahedral layers.^32^ In PLS materials, additional oxygen atoms cause a breaking of the octahedral linkages resulting in gaps between the octahedral layers. In the case of an A_2_B_2_O_7_ system, these gaps occur between every fourth layer of octahedra (Figure 8a). In the present case, the layers are stacked in the *b*-direction, and in space group *Cmc*2_1_, the gap can be defined as the distance in the *b*-direction between adjacent O3 atoms with the size of the perovskite block defined as the distance between O3 atoms in the first and fourth layers. Similarly, the degree of distortion away from centrosymmetric symmetry can be monitored through the distance (Δ*L*) in the *b*-direction between O3 and O7 atoms on the same octahedron. The thermal variation of these three quantities is given in Figure 8c–e. On heating, the width of the perovskite block increases with increasing temperature up to 600 °C but remains almost constant above this temperature. In contrast, the gap distance shows no significant change up to around 650 °C but then increases with increasing temperature above this point. Δ*L* remains almost constant up to ca. 500 °C but then decreases sharply with increasing temperature (Figure 8e). At 700 °C, Δ*L* is close to zero, which would correspond to the equivalence of O3 and O7 atoms as in the centrosymmetric space group. These results are consistent with a gradual change in the structure above ca. 600 °C from a polar phase in space group *Cmc*2_1_ to a nonpolar phase in space group **Cmcm**.

The changes seen in Figure 8 are clearly associated with a high-temperature phase transition as indicated by the DSC data (Figure 3) and the anomaly observed in the dielectric spectrum (Figure 2) at around 750 °C. At room temperature, when STLT32 exists as the polar phase, the tilting of the BO_6_ octahedra and the resulting distortion lead to a spontaneous polarization. With increasing temperature, the size of the perovskite block increases due to thermal expansion, while the size of the gap remains fairly constant as does the degree of distortion, Δ*L*. At around 550 °C, Δ*L* starts to decrease significantly as the polar distortion begins to diminish before the transition to a centrosymmetric structure at *T*_C_. This decrease in distortion compensates for the thermal expansion in the perovskite block such that the width of the block remains constant above ca. 600 °C, while the size of the gap increases with increasing temperature above this point.

**Figure 9:**
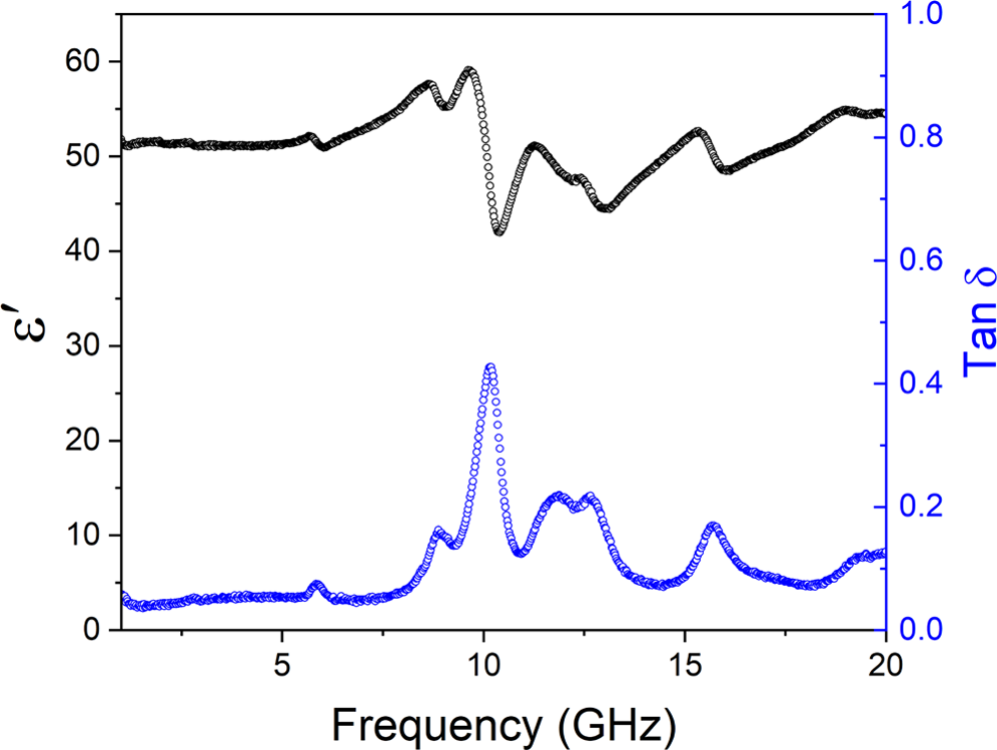
Observed dielectric permittivity and loss tangent at microwave frequencies of an STLT32 ceramic with a 5 mm transmission line deposited on its surface.

Figure 9 shows the measured data for permittivity and loss tangent for an STLT32 pellet with a 5 mm transmission line deposited on its surface and covers the frequency range 2–18 GHz. Below 10 GHz, the real part of the permittivity remains stable across the band with a value of around 51 and a loss tangent of 0.03–0.05, consistent with the lower frequency values from Figure 2. At frequencies above 10 GHz, some resonance peaks are observed in the dielectric permittivity plot with corresponding dielectric loss peaks. Permittivity and loss peaks are also observed with a 1 mm transmission line over the frequency range from 8 to 11 GHz (Figure S11). These anomalies are attributed to the geometry of the transmission line on the sample rather than an intrinsic property of the material itself.^33^ Thus, STLT32 exhibits stable dielectric properties over a large range of frequencies up to the microwave range. This suggests that the material could be a candidate for integration in future 5G wireless systems.

## 4 Conclusions

The PLS materials Sr_1.68_La_0.32_Ta_1.68_Ti_0.32_O_7_ (STLT32), Sr_1.64_La_0.36_Ta_1.64_Ti_0.36_O_7_ (STLT36), and Sr_1.85_Ca_0.15_Ta_2_O_7_ (SCT15) were synthesized by solid-state methods and show AFE-like electrical behavior. In the case of STLT32, this AFE-like behavior occurs despite neutron diffraction and PFM images confirming its structure to be polar at room temperature. Remarkably, STLT32 exhibits double *P–E* hysteresis loops at low and high electric fields over a wide temperature range (ambient to 200 °C), the first time such behavior has been observed in a PLS system. Four current peaks in the *I–E* data are likely to be associated with the electric field-induced phase transition between weakly polar and more strongly polar phases. Two anomalies are observed in the dielectric spectrum of STLT32 at ca. 220 and 750 °C with the latter feature confirmed by DSC. A subtle phase transition at ca. 250 °C is also indicated in variable temperature neutron diffraction analysis, while the higher-temperature transition, at around 750 °C, is found to involve a change in symmetry from polar to nonpolar structures. This transition is associated with a gradual reduction of octahedral tilting in the perovskite blocks and an increase in the interlayer gap size. A stable dielectric permittivity of ca. 51 was seen in an STLT32 ceramic up to microwave frequencies with low dielectric loss. These new findings in the dielectric properties and structural behavior of a PLS material could lead to an expansion of their potential applications.

## Supporting Information Available

Additional characterization data including fitted XRD profiles, SEM images, elevated temperature *P*–*E*/*I*–*E* loops, DC field dependence of dielectric permittivity, thermal variation of the *c*/*a* lattice parameter ratio, dielectric behavior at microwave frequencies, crystal and refinement parameters, and refined structural parameters (PDF)
