# Supplementary material for: Antiferroelectric-like Behavior in a Lead-Free Perovskite Layered Structure Ceramic
Source: Inorg Chem. 2022 Dec 6;61(50):20316–25. doi: 10.1021/acs.inorgchem.2c02726 (PMC9768747; doi:10.1021/acs.inorgchem.2c02726)
Supplement: Supplementary file 1 — ic2c02726_si_001.pdf [file ic2c02726_si_001.pdf]

Supporting Information

Antiferroelectric-like behavior in a lead-free perovskite layered structure ceramic

Hangfeng Zhang,[1] A. Dominic Fortes,[2] Henry Giddens,[1] Theo Graves Saunders,[1] Matteo Palma,[3] Isaac Abrahams,[3*] Haixue Yan,[4*] Yang Hao[1*]

[1]School of Electronic Engineering and Computer Science, Queen Mary University of London, Mile End Road London E1 4NS, UK

[2]STFC ISIS Facility, Rutherford Appleton Laboratory, Chilton Didcot, Oxfordshire, OX11 OQX, UK.

[3]Department of Chemistry, Queen Mary University of London, Mile End Road, London E1 4NS, UK.

[4]School of Engineering and Materials Sciences, Queen Mary University of London, Mile End Road, London E1 4NS, UK.

*Corresponding Authors

I. Abrahams tel: +44 207 882 3235 email: i.abrahams@qmul.ac.uk

H. Yan tel: +44 207 882 5164 email: h.x.yan@qmul.ac.uk

Y. Hao tel: +44 207 882 5341 email: y.hao@qmul.ac.uk

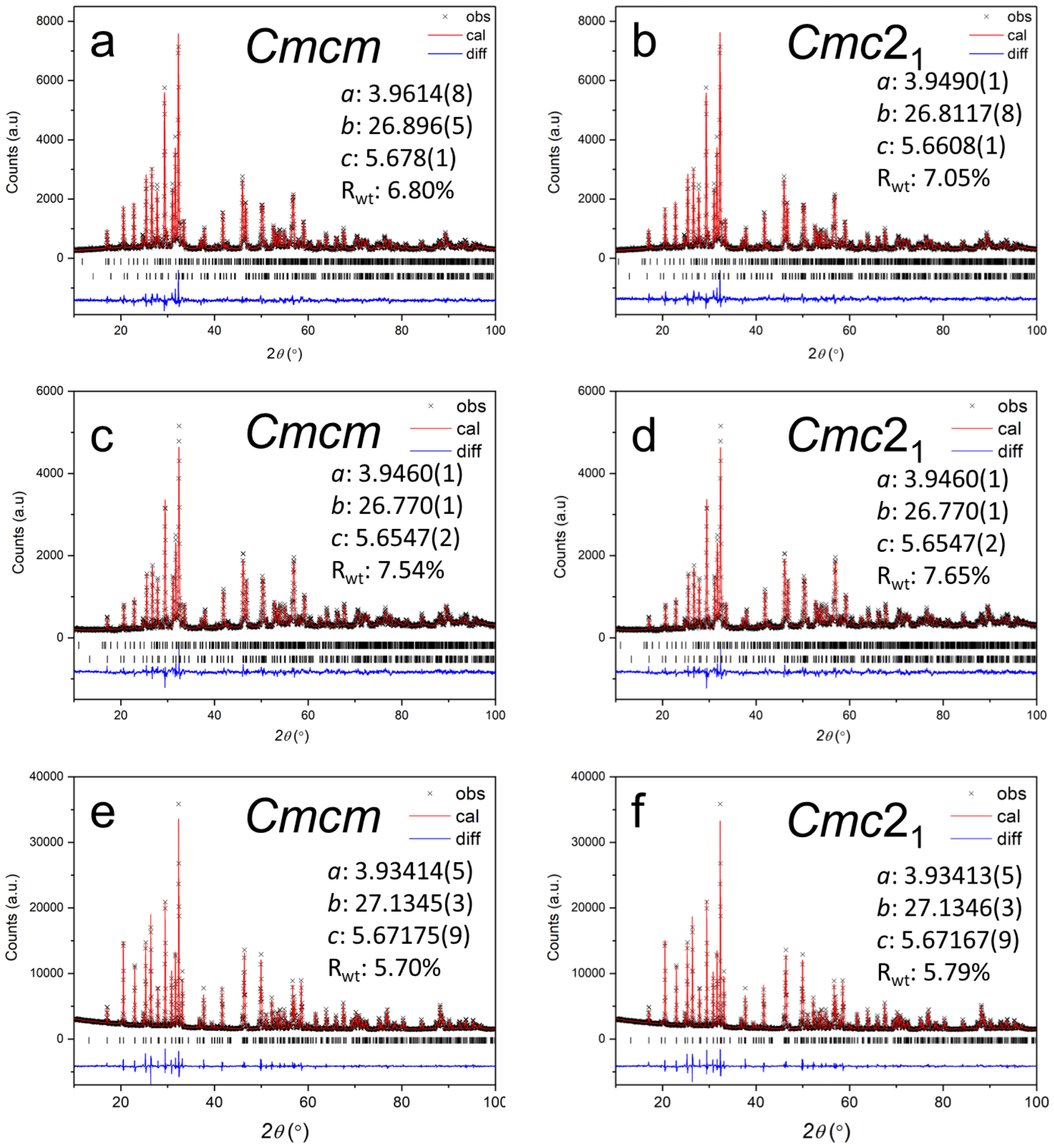

Figure S1. Fitted X-ray powder diffraction profiles for (a,b) STLT32, (c,d) STLT36 and (e, f) SCT15 using *Cmcm* and *Cmc*2_1. A 5-layer structure in space group *Pnmm* was included as a secondary phase in the refinements for the two STLT compositions. Reflection positions are indicated by markers.

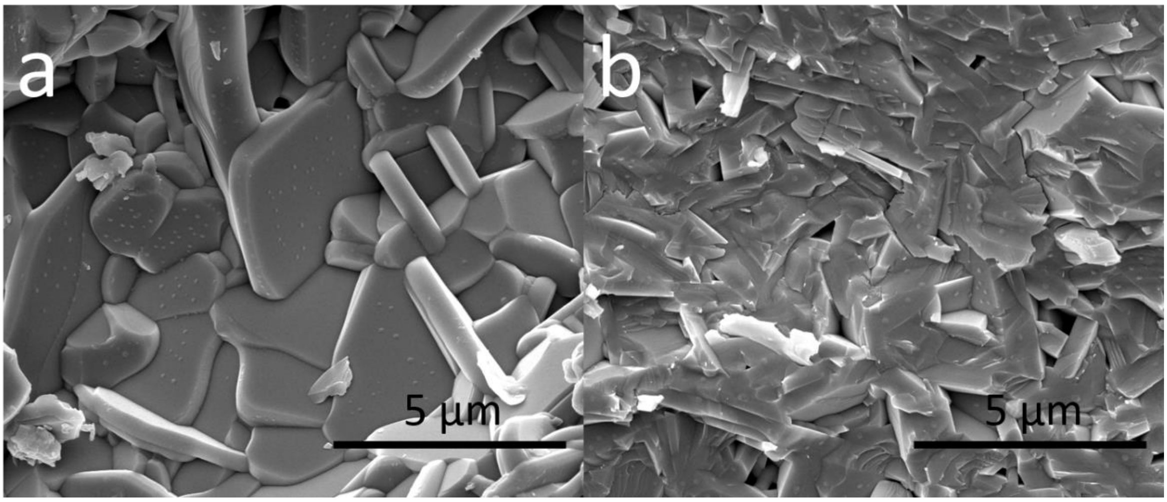

Figure S2. SEM images of sintered STLT32 pellet: (a) as sintered surface and (b) cross-section.

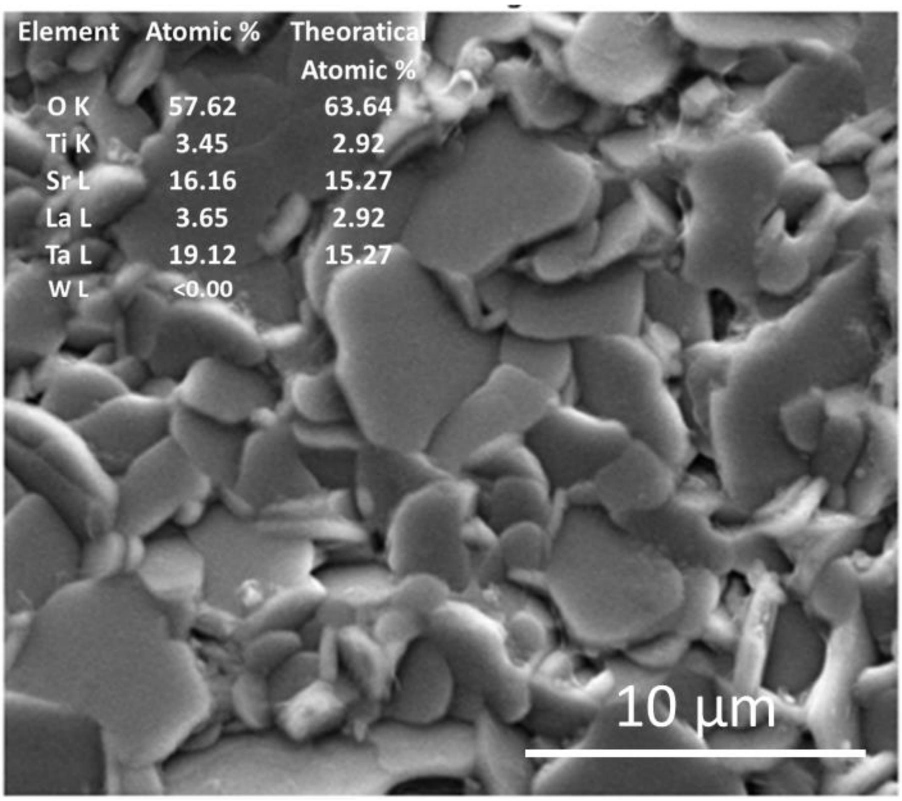

Figure S3. SEM and summary of EDX analysis results for an STLT32 sample.

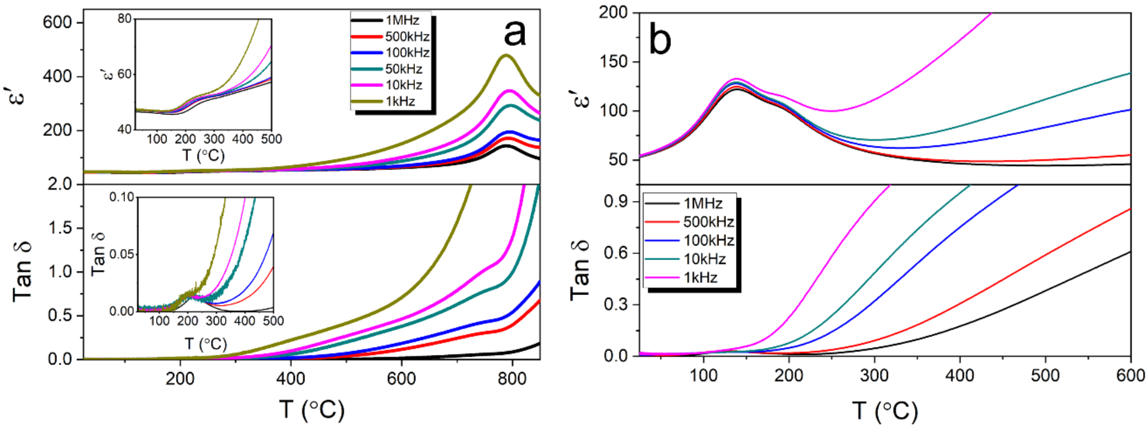

Figure. S4, Thermal dependencies of dielectric permittivity and loss tangent for (a) STLT36 and (b) SCT15.

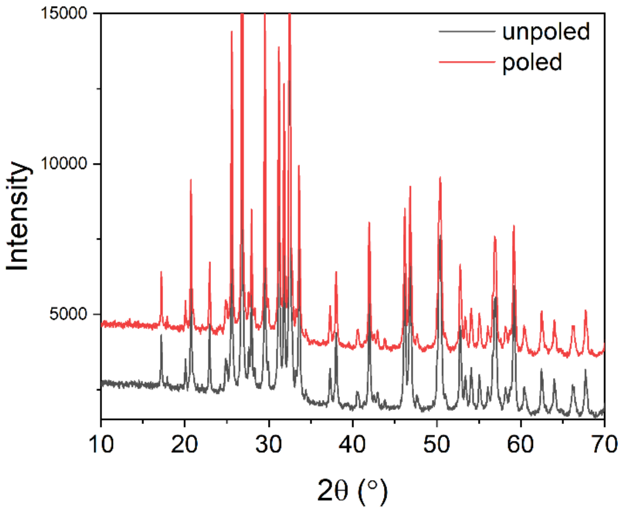

Figure S5. XRD patterns of an unpoled and poled STLT32 ceramic sample.

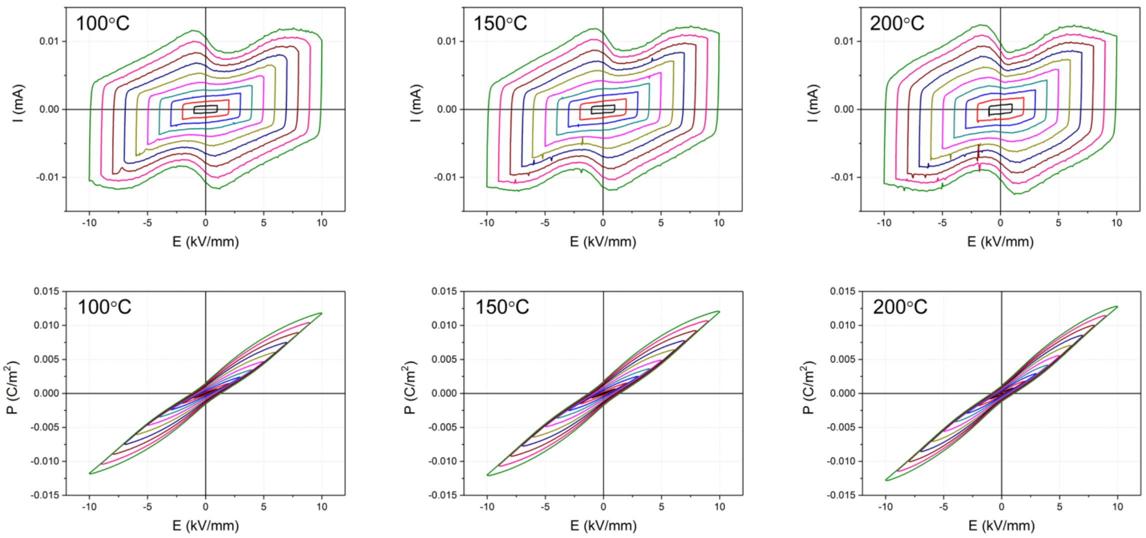

Figure S6. *I-E*/*P-E* loops of STLT32 at selected temperatures.

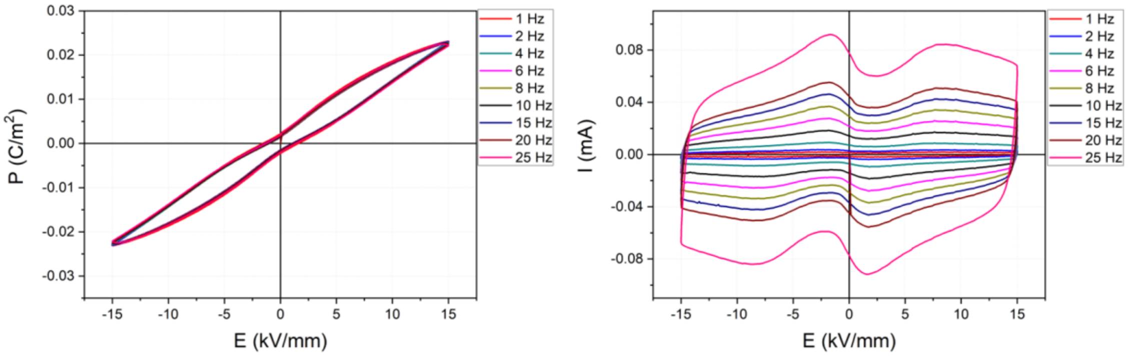

Figure S7: *P-E* (left) and *I-E* (right) loops for STLT32 at selected frequencies.

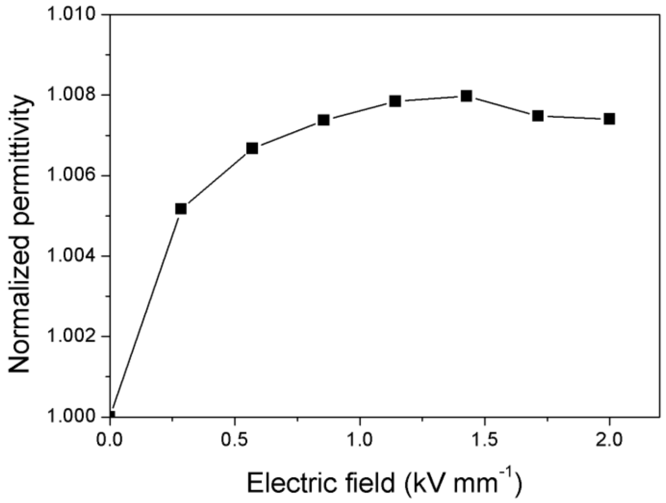

Figure S8. Variation of normalized dielectric permittivity ($\varepsilon/\varepsilon_0$, where ε_0 is the permittivity at 0 V) with DC electric field in STLT32.

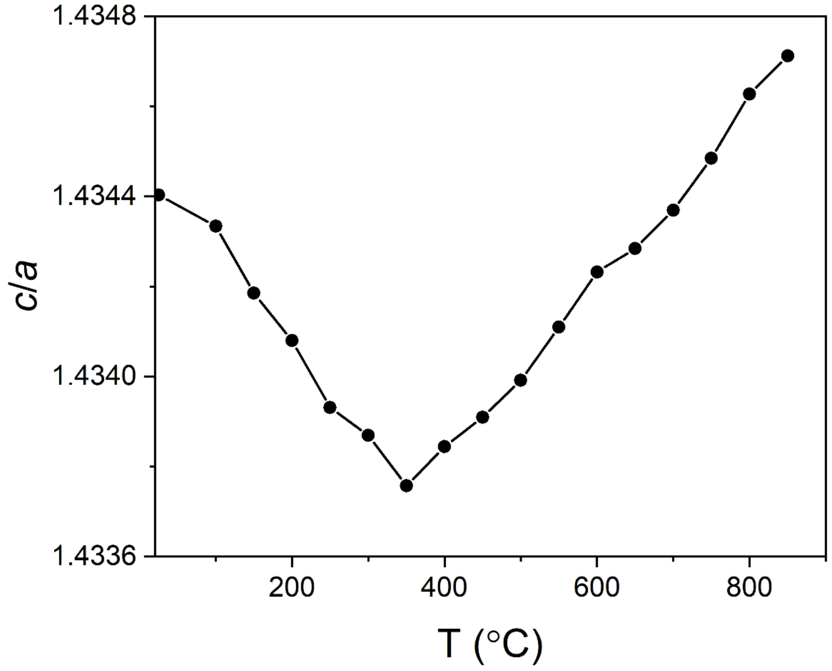

Figure S9. Thermal variation of the *c*/*a* lattice parameter ratio in STLT32.

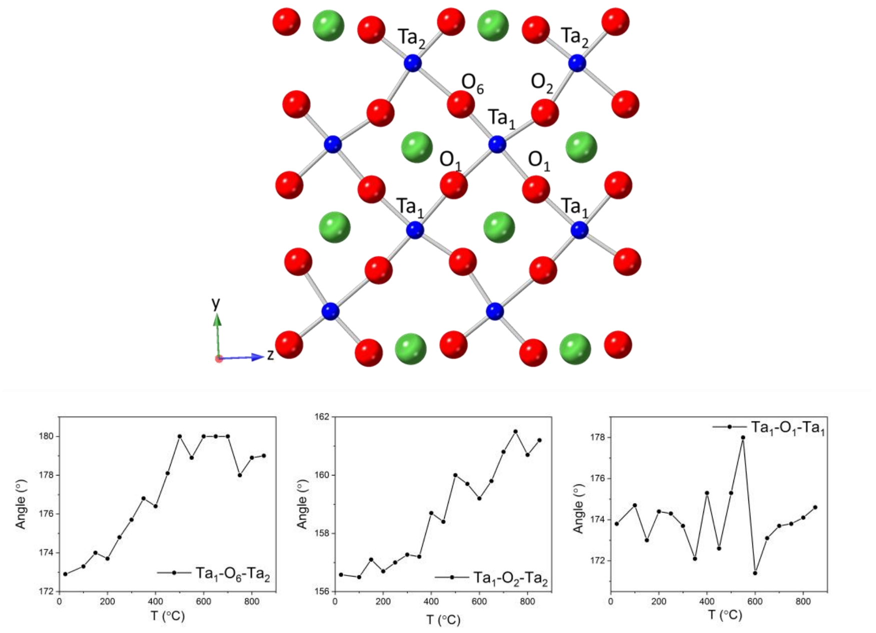

Figure S10, Thermal variation of Ta-O-Ta bond angle in STLT32.

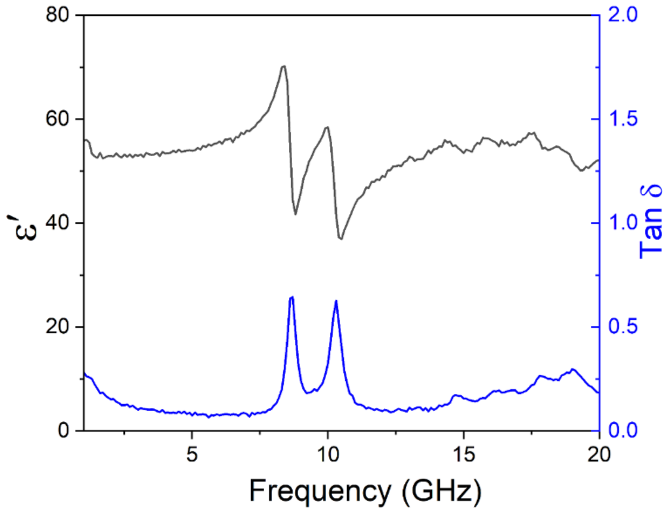

Figure S11. Dielectric permittivity and loss tangent of STLT32 pellet with 1 mm transmission line measured at microwave frequencies.

Table S1. Crystal and refinement parameters for STLT32 at room temperature based on fits to high-resolution neutron diffraction data. Estimated standard deviations are given in parentheses.

**Space group**	**(a) *Cmc*2₁**	**(b) *Cmcm***
**Weight%**	89.54(5)	85.2(3)
**Unit cell dimensions**	*a* = 3.94671(5) Å<br>*b* = 26.8262(4) Å<br>*c* = 5.66118(8) Å	*a* = 3.94642(9) Å<br>*b* = 26.8212(6) Å<br>*c* = 5.6605(1) Å
**Volume (Å^3)**	599.38(2)	599.19(1)
**Z**	4	4
**D_{calc} (g cm^{-3})**	6.904	6.906
**Space group**	***Pmnm***	***Pmnm***
**Weight%**	10.4(1)	14.7(3)
	a = 5.6465(3) Å<br>*b* = 32.528(1) Å<br>*c* = 3.9404(1) Å	*a* = 5.6442(4) Å<br>*b* = 32.518(2) Å<br>*c* = 3.9425(3) Å
**Volume (Å^3)**	723.76(5)	723.61(8)
**Z**	2	2
**D_{calc} (g cm^{-3})**	6.799	6.801
**R-factors[α]**	R_{wp} = 0.0235<br>R_p = 0.0214<br>R_{ex} = 0.0102<br>R_F^2 = 0.0780	R_{wp} = 0.0406<br>R_p = 0.0337<br>R_{ex} = 0.0102<br>R_F^2 = 0.1540
**No. of variables**	79	65
**No. of profile points**	4609	4609

Table S2. Refined structural parameters for STLT32 at room temperature based on fits to high-resolution neutron diffraction data using *Cmc*2_1 and *Cmcm* models. Estimated standard deviations are given in parentheses.

***Cmc*2_1**

Atom	Site	*x*	*y*	*z*	Occ.	U_{iso} (Å^2)
**Sr1/La1**	4a	0.0	0.44865(15)	0.7612(13)	0.68/0.32	0.0237(5)
**Sr2**	4a	0.0	0.29263(11)	0.2234(10)	1.00	0.0237(5)
**Ta1/Ti1**	4a	0.5	0.44503(16)	0.25	0.84/0.16	0.0053(3)
**Ta2/Ti2**	4a	0.5	0.34080(12)	0.7356(14)	0.84/0.16	0.0053(3)
**O1**	4a	0.5	0.50273(29)	0.4853(14)	1.00	0.0244(9)
**O2**	4a	0.0	0.40446(17)	0.5396(13)	1.00	0.0102(8)
**O3**	4a	0.5	0.29805(24)	0.4838(12)	1.00	0.0370(16)
**O4**	4a	0.0	0.44993(19)	0.2758(10)	1.00	0.0179(9)
**O5**	4a	0.5	0.34767(19)	0.7736(12)	1.00	0.0275(11)
**O6**	4a	0.5	0.39361(26)	0.0282(13)	1.00	0.0434(18)
**O7**	4a	0.5	0.28778(19)	0.9681(10)	1.00	0.0128(9)

Cmcm

Atom	Site	*x*	*y*	*z*	Occ.	U_{iso} (Å^2)
**Sr1**	4c	0.0	0.29338(18)	0.25	1.00	0.0296(8)
**Sr2/La2**	4c	0.0	0.44640(21)	0.75	0.68/0.32	0.0296(8)
**Ta1/Ti1**	4c	0.5	0.34076(17)	0.75	0.84/0.16	0.0055(5)
**Ta2/Ti2**	4c	0.5	0.44485(23)	0.25	0.84/0.16	0.0055(5)
**O1**	8f	0.5	0.29194(14)	0.5108(7)	1.00	0.0246(8)
**O2**	4c	0.0	0.34560(30)	0.75	1.00	0.0429(19)
**O3**	8f	0.5	0.40220(19)	0.5319(10)	1.00	0.0527(17)
**O4**	4c	0.0	0.45323(22)	0.25	1.00	0.0195(12)
**O5**	4a	0.5	0.5	0.5	1.00	0.0162(10)

Table S3, R-factors and refined unit cell parameters for refinements of the STLT32 structure using neutron diffraction data recorded at selected temperatures. Estimated standard deviations are given in parentheses.

Temperature/°C	*a*/Å	*b*/Å	*c*/Å	V/Å^3	R_{wp}
25	3.94671(5)	26.8262(4)	5.66118(7)	599.38(2)	0.0235
100	3.94834(7)	26.8370(5)	5.6632(1)	600.08(3)	0.0331
150	3.95092(7)	26.8534(6)	5.6663(1)	601.17(3)	0.0329
200	3.95383(7)	26.8705(6)	5.6701(1)	602.40(3)	0.0317
250	3.95635(7)	26.8869(6)	5.6731(1)	603.47(3)	0.0320
300	3.95909(4)	26.9072(3)	5.67683(7)	604.74(2)	0.0191
350	3.96044(7)	26.9197(6)	5.6783(1)	605.39(3)	0.0312
400	3.96215(7)	26.9377(6)	5.6811(1)	606.35(3)	0.0315
450	3.96386(7)	26.9563(5)	5.6838(1)	607.32(3)	0.0311
500	3.96522(7)	26.9750(5)	5.6860(1)	608.19(2)	0.0309
550	3.96672(6)	26.9947(5)	5.68872(9)	609.15(2)	0.0308
600	3.96817(6)	27.0153(5)	5.69128(9)	610.11(2)	0.0307
650	3.96970(6)	27.0355(5)	5.69368(9)	611.06(2)	0.0312
700	3.97118(6)	27.0569(4)	5.69614(8)	612.04(2)	0.0305
750	3.97272(6)	27.0778(4)	5.69881(8)	613.03(2)	0.0315
800	3.97411(5)	27.0911(4)	5.70137(8)	613.83(2)	0.0312
850	3.97565(3)	27.1055(2)	5.70391(5)	614.66(1)	0.0196

Reference

1. Larson. A. C,; Dreele. R. B (1987) *Los Alamos National Laboratory Report*. No. LAUR-86-748.
